# What makes mentors thrive? An exploratory study of their satisfaction in undergraduate medical education

**DOI:** 10.1186/s12909-024-05344-y

**Published:** 2024-04-04

**Authors:** Elise Pauline Skjevik, Edvin Schei, J. Donald Boudreau, Arne Tjølsen, Unni Ringberg, Abraham Fuks, Monika Kvernenes, Eirik H. Ofstad

**Affiliations:** 1 Department of Community Medicine, Faculty of Health Sciences, UiT The Arctic University of Norway, Tromsø, 9037 Norway; 2 Department of Global Public Health and Primary Care, Faculty of Medicine, The University of Bergen, Bergen, Norway; 3 Institute of Health Sciences Education, Faculty of Medicine and Health Sciences, McGill University, Montreal, Canada; 4 University of Notre Dame, Sydney, Australia; 5 Department of Biomedicine and Center for Medical Education, Faculty of Medicine, The University of Bergen, Bergen, Norway; 6 Department of Medicine, Faculty of Medicine and Health Sciences, McGill University, Montreal, Canada; 7 Department of Clinical Medicine, Center for Medical Education, Faculty of Medicine, The University of Bergen, Bergen, Norway; 8 Department of Clinical Medicine, UiT The Arctic University of Norway, Tromsø, Norway

**Keywords:** Mentoring, Group mentorship, Medical education, Satisfaction, Rewards

## Abstract

**Background:**

Mentoring medical students with varied backgrounds and individual needs can be challenging. Mentors’ satisfaction is likely to be important for the quality and sustainability of mentorships, especially in programs where the mentor has responsibility for facilitating a group of mentees. However, little is known about what influences mentors’ satisfaction. The aim of this study was to measure mentors’ self-reported satisfaction with the mentoring experience and to explore associations between satisfaction and its putative factors.

**Methods:**

An online survey was sent out to all physician mentors in each of the three mentorship programs (UiT The Arctic University of Norway, the University of Bergen, and McGill University, graduation years 2013–2020, *n* = 461). Data were analyzed by descriptive statistics, dimension reduction, and linear regression.

**Results:**

On a scale from 1 to 5, mean mentor satisfaction score at two Norwegian and one Canadian medical school was 4.55 (95% CI 4.47, 4.64). In a multilevel multivariate regression analysis, two predictors were significantly associated with mentors’ satisfaction: (1) the perception that students found the group meetings valuable (β = 0.186, 95% CI 0.021, 0.351, *p* = 0.027) and (2) mentors’ perceived rewards (β = 0.330, 95% CI 0.224, 0.437, *p* < 0.001). Perceived rewards included experiencing gratifying relationships with students, and mentors’ perception of self-development.

**Conclusions:**

In this study, mentors appeared to be highly satisfied with their mentoring functions. Our findings suggest that mentors’ overall satisfaction is closely linked to their experiences of fulfilling mentor-student relationships and personal and professional development. Interestingly, and perhaps contrary to commonly held assumptions, we found no association between mentor satisfaction and financial compensation. Furthermore, satisfaction was not associated with the provision of pre-assigned topics for discussions for mentor group meetings. We propose that the mentors’ experienced psycho-social rewards, and their competence in establishing well-functioning group dynamics, should be areas of focus for faculty development.

**Supplementary Information:**

The online version contains supplementary material available at 10.1186/s12909-024-05344-y.

## Background

Mentoring, notably in formats where mentors are given responsibilities for a group of mentees, is increasingly used in medical education [1]. Mentorships offer rich opportunities for building relationships, professional identity formation, and for the promotion of reflection [2–4]. Such outcomes are favored through the nurturing of shared goals, and in psychologically safe contexts where students and mentors have diverse backgrounds and experiences [1, 5]. Mentoring medical students in groups may also be an effective way of making mentoring available to a large number of students [6].

Several studies assessing mentorship programs, mostly one-on-one, have found that students report positive effects, e.g. increased personal support [7–9], professional growth and improved satisfaction with the medical education experience [1, 7, 10–13]. Mentors have been shown to benefit as well, through enhanced personal development [14, 15] and improved clinical skills, such as listening and communication [11].

Desirable mentor characteristics are described in the medical literature [6, 16–19]. Mentors should be active listeners. They must engage in and understand messages that are not explicitly communicated (e.g. body language, facial expressions) [16]. Student-centeredness and a willingness to develop both personally and professionally are other desirable mentor qualities [16, 17]. A good mentor has been described as enthusiastic, selfless, and knowledgeable [17, 20].

Mentors in medical education are often clinicians or medical teachers who adopt the role with minimal preparation or training in mentoring and small group facilitation [3, 21]. Being a mentor for a group of students with varied backgrounds and individual needs can be challenging [22, 23]. One of the key impediments to the establishment of well-functioning mentorship groups is uncertainty on the part of mentors regarding the nature and goals of their role [1]. Mentors thus need clearly defined expectations of mentoring, and support in developing listening and feedback skills [24].

There is a gap in the literature as to which factors are associated with mentors’ satisfaction in group-based programs. The purpose of our research was, using a survey instrument, to measure mentors’ self-reported satisfaction with the mentoring experience and to explore associations between satisfaction and its putative factors. Mentors’ satisfaction may be decisive for the pedagogical quality and the sustainability of group mentorship programs in medical education [13, 25].

## Methods

### Context

The present study is part of a larger multi-center research project (called CanNorMent), a collaborative investigation of group mentorship programs at three medical schools: UiT the Arctic University of Norway (UiT), the University of Bergen (UiB), Norway, and McGill University, Canada.

They are all longitudinal and mandatory mentorships in which pairs of mentors facilitate discussions within a group of medical students. The programs share a focus on establishing a safe environment where students, in dialogue with peers and mentors, can: (1) share thoughts and discuss the challenging experiences and processes of medical studies that may contribute to professional identity formation, and (2) reflect on patient encounters and the goals of medicine. The characteristics of the three group mentorship programs are summarized in Table 1.

**Table 1:** Characteristics of the three mentorship programs (at the time of the study)

	McGill University, Canada	University of Bergen, Norway	UiT The Arctic University, Norway
**Duration of medical school**	4 years	6 years	6 years
**Students per year**	180	150	100
**Mentor program**	MS1-MS4	MS2-MS5	MS1-MS4 and MS6
**Mentor program established**	2005	2014	2012
**Group size**	6 students	8 students	8 students
**Meetings**	4–8 meetings a year. Attendance at 75% of meetings is compulsory.	5 meetings a year. Attendance at 75% of meetings is compulsory.	4 meetings a year + 1 one-on-one meeting (30 min). Attendance at 75% of meetings is compulsory.
**Mentors**	1 physician and 1 senior (MS3 or MS4) student.	2 physicians.	2 physicians.
**Content emphasis**	The content is driven by the students’ preferences. Mentors have backup content if needed.	The content is driven by the students’ preferences. Mentors are oriented about person-centred medicine and medical professionalism.	Every group meeting has a predetermined topic and students prepares tasks to present and discuss.
**Criteria for mentor recruitment**	Recruit mentors from all the specialties in medicine; approximately 1/3 are from the Department of Family Medicine. Strives to recruit mentors who are seeing patients in their daily work.	Recruit mentors through informal networks and snowballing techniques. Recruitment is purposely seeking to enroll physicians and pairing mentors with the aim of maximum variation.	Head of institutes recruit mentors. Both mentors must be physicians by education; otherwise, there are no criteria for being a mentor.
**Faculty development**	3 workshops per year.	One introductory seminar and 2–3 annual seminars.	One introductory seminar and one annual seminar.
**Additional financial compensation**	Yes.	Yes.	No.

The number following MS denotes the year of the program (e.g., MS-1 refers to a 1^st^-year medical student)

*MS* Medical student

### Survey instrument and participants

The survey categories, items and responses are presented in Appendix 1. At the time of the study, we were not able to find a validated questionnaire suitable for our research question. Consequently, with inspiration from Stenfors’ research [5] and Prosser and Trigwells’ approaches to a teaching inventory adapted to mentoring [26], we developed a survey using a combination of closed (*n* = 28) and open-ended questions (*n* = 8). The language was English. It was pilot tested on three mentors in both Canada and Norway, which led to minor adjustments of some questions. The mentor pairs at UiT and UiB were both physicians, whereas mentor pairs at McGill consisted of a physician with a senior medical student as co-mentor. Thus, the questions regarding co-mentorship were different in the Norwegian and Canadian surveys.

All physician mentors who had participated in each of the mentorship programs (graduation years 2013–2020, *n* = 461) were invited to participate. No incentives were offered. The study population at each site was as follows: 114 mentors at UiT; 123 at UiB; and 224 at McGill. Since mentors at McGill accounted for approximately 50% of the responses, a chi-square test was conducted after dichotomizing the outcome variable. The distribution of the outcome did not significantly differ by university ((χ² (2, *N* = 272) = 1.78, *p* = 0.41)), thus it was appropriate to aggregate the responses from all the participants. As the survey aimed to map specific topics concerning group mentoring, some survey items were not relevant for the aim of this specific paper and hence are not discussed here.

The study was approved by the Institutional Review Board at McGill University (Study Number A03-B16-17B) and the Norwegian Centre for Research Data (ID 53,715). The survey was distributed by e-mail to the mentors using the platform SurveyXact, with two reminders. Responses were stored on a high-security server at UiB where all analyses were carried out on encrypted files, with personal information (name and e-mail address) detached.

### Statistics

In this study, the primary outcome variable was mentors’ satisfaction, derived from the question “If you consider the totality of your experience of being a mentor, how do you like it?”. The responses ranged from ‘I dislike being a mentor’=1 to ‘I like being a mentor’=5. Statistical analyses were performed using IBM SPSS version 29. Descriptive statistics were conducted for respondents’ characteristics and several of the questions in the core survey instrument, including means and distributions.

### Factor analyses

The respondents rated their agreement with nine statements describing how they approached group mentoring. The respondents also rated their agreement with seven items of rewards from mentoring and three items regarding their perception of student resistance in the groups. Several of the items in each of these categories (“Mentoring approach”, “Rewards”, and “Student resistance”, see Appendix 1) were highly intercorrelated, thus factor analyses (dimensional reduction; varimax rotated) were used to develop indices that could be used as independent variables in the analyses of mentors’ satisfaction. The Kaiser-Meyer-Olkin (KMO) measure of sampling adequacy along with Bartlett’s test of sphericity were performed to decide the appropriateness of the factor analyses [27, 28]. For details regarding items and factor loadings in the exploratory analyses, see Appendix 2.

For the mentors’ approach to group mentoring (KMO = 0.75), an exploratory factor analysis was conducted, after which three of the items were removed due to low loadings on the first factor. A confirmatory factor analysis of the six remaining items yielded one factor with eigenvalue larger than 1, explaining 44.1% of the variance (Cronbach’s α = 0.77). The index was labeled ‘Engaging mentoring approach’.

For the group of reward items (KMO = 0.81), one item concerning financial rewards was removed due to minimal loading to the first factor in an exploratory factor analysis. The confirmatory analysis of the remaining six items lead to a factor explaining 58.8% of the variance (Cronbach’s α = 0.84). This index was labeled ‘Perceived mentoring rewards’.

Lastly, a factor analysis was done on the three items regarding mentors’ perception of student resistance in the groups (KMO = 0.61). In this case, only one factor was extracted, explaining 61.1% of the variance (Cronbach’s α = 0.67). This index was labeled ‘Perceived student resistance’.

### Multilevel linear regression

As this was a multi-institutional study, there was a need to explore if and how context affected the findings. Mentors’ experiences may be shaped by the social context in which they occur. Multilevel modelling provides a framework to account for such complex data structures [29]. Consequently, a multilevel multivariate linear analysis was completed to explore the association between the primary outcome and the level one (individual) and level two (university) predictors. The intraclass correlation coefficient (ICC) [30] with mentors’ satisfaction as a dependent variable and universities as random intercept was 0.034. The design effect (DEFF) was above the criteria of 2, indicating a need for multilevel modelling [29].

First, a multilevel univariate linear regression analysis was performed with each of sixteen predictor variables and mentors’ satisfaction as the dependent variable (Table 2). Second, only statistically significant predictors were included in the final multilevel multivariate linear regression analysis, in addition to age and gender. The significance threshold for the regression analyses was set at 5% (*p* < 0.05). A stepwise regression method was used to fit the final model. Prior to modelling, the Variance Inflation Factor (VIF) [31] was utilized to explore multicollinearity. This test indicated that predictor multicollinearity was not a problem (VIF mean = 1.672).

**Table 2:** Predictor variables for Mentor’s satisfaction; on University level (Level 2) and Mentor level (Level 1) by univariate multilevel linear regression analysis

	Category	Items - questions in questionnaire or results of factor analysis	β	*P*-value*	95% CI
**Level 2 predictors**	Mentor recruitment	Did you volunteer to become a mentor, or is it mandatory in your job?	-0.211	0.008**	[-0.367,-0.055]
	Structured meetings	Based on information on the three programs	0.056	0.299	[-0.049,0.158]
	Additional financial compensation	Based on information on the three programs	0.056	0.299	[-0.049,0.158]
**Level 1 predictors**	Mentor characteristics	Age	0.008	0.487	[-0.049,0.102]
		Gender	0.002	0.908	[-0.161,0.181]
		What is the nature of your current work?	-0.013	0.600	[-0.161,0.093]
		For how many years, in total, have you been a mentor of medical students?	0.012	0.023**	[0.002,0.026]
	Mentor support	I find it unclear what the mentor program’s expectations are	0.185	< 0.001**	[-0.260,-0.110]
		I find it difficult to fulfil the program’s expectations	0.184	< 0.001**	[-0.269,-0.107]
		What is your opinion about the quality of the training provided, including workshops and information meetings, to help mentors?	0.247	< 0.001**	[0.148,0.342]
		What is your opinion about the quality of the written material on mentoring and the mentor program, provided to you as a mentor?	0.187	< 0.001**	[0.081,0.289]
	Student perspective	The students seem to find the group meetings valuable	0.466	< 0.001**	[0.351,0.580]
		The students in my group have lots of ideas for the group process and discussions	0.259	< 0.001**	[0.180,0.338]
	‘Engaging mentoring approach’	Factor containing six items that illustrates an engaging way to be a mentor	0.228	< 0.001**	[0.148, 0.310]
	‘Perceived rewards’	Factor containing six items that illustrates potential rewards of being a mentor	0.428	< 0.001**	[0.330, 0.473]
	‘Perceived student resistance’	Factor containing three items that illustrates students’ resistance in the groups	-0.238	< 0.001**	[-0.331,-0.144]

*P-value in the univariate analyses

**Significant at *p* < 0.05

## Results

The characteristics of the mentors are summarized in Table 3. The overall response rate was 59% (*n* = 272/461). 117 mentors were female (43%) and 153 were male (57%). The percentage of female mentors was higher at McGill and UiB than at UiT (45% and 49% versus 32%, respectively). The mentors were categorized into four groups by age: <40 years (21%), 40–49 years (28%), 50–59 years (23%) and > = 60 years (28%). At McGill and UiB, a larger percentage of mentors were below 50 years of age than at UiT (51% and 38% versus 13%, respectively). Most of the mentors responded that their nature of work was mostly clinical (69%), while a minority had some or no clinical responsibilities (21% and 10%, respectively).

**Table 3:** Mentors’ characteristics

		McGill University, Canada	UiB, Norway	UiT, Norway	Total
**Characteristics**^**a**^					
**Responders, n (% of invited)**		137 (61)	75 (61)	60 (53)	272 (59)
**Gender, %**	Female	45	49	32	43
	Male	54	51	68	57
**Age (years), %**	< 40	13	40	12	21
	40–49	38	23	13	28
	50–59	20	19	33	23
	60+	28	18	42	28
**Nature of work, %**	Mostly clinical	85	67	33	69
	Some clinical	13	18	45	21
	No clinical	1	14	17	10

^a^Two participants did not answer any of these questions regarding characteristics

Responses to the following set of questions in the survey instrument are presented in Table 4: mentors’ satisfaction, questions addressing the mentors’ approach to group mentoring and questions regarding rewards. The distribution of mentors’ satisfaction was skewed towards positive assessment; mean score across the universities was 4.55 (95% CI 4.47, 4.64). The two highest scores among the questions regarding mentors’ approach to mentoring, were “sharing what it means to be a doctor” (mean 4.36, 95% CI 4.28, 4.43) and “sharing experiences of doubt and uncertainty” (mean 4.29, 95% CI 4.31, 4.44).

**Table 4:** Mentors’ satisfaction, Mentoring Approach and Reward Items, distribution of Likert scale responses. Horizontal bars: Likert score 1; (strongly disagree) dark blue, 2; orange, 3; grey, 4; yellow, 5; (strongly agree) light blue

Survey item	Mean	Distribution of responses across 1–5 Likert scale
Mentors’ satisfaction: If you consider the totality of your experience of being a mentor, how do you like it?	4.55	
**Mentoring approach**		
As a mentor I:		
- Answer questions and provide knowledge	3.90	
♦ Share what it means to be a doctor	4.36	
- Listen to students without offering advice	2.96	
♦ Stimulate collaboration and relationships within the group	4.04	
♦ Am a role model for the students	4.17	
- Provide career counselling	3.48	
♦ Take an interest in students’ personal development	4.15	
♦ Share my experiences of doubt and uncertainty	4.29	
♦ Share my attitudes and judgments concerning values and dilemmas in medicine	3.35	
**Rewards**		
* Being a mentor has helped me become better at what I do professionally	3.73	
* I learn a lot from discussing with students	3.70	
* The preparation offered to all mentors gives me new knowledge	3.20	
* The relationships with the students are gratifying	4.16	
* Mentoring makes me more proud of being a physician	3.66	
* Mentoring allows me to explore what it means to be a “good doctor”	3.88	
- Mentoring provides financial rewards	1.32	

♦= Items representing result from factor analysis: ‘Engaging mentoring approach’

*= Items representing result from factor analysis: ‘Perceived Rewards’

The ’Engaging mentoring approach’ index represents a summary of the items where the respondents express their approach to mentoring. Six of these items are correlated, so that they to a large extent show aspects of the same approach or attitude. Therefore, it is appropriate to use a common index for the mentoring approach, as it is described in the statistics section. The index thus illustrates mentoring approaches related to sharing of experiences, attitudes and meaning, role modelling, group facilitation (stimulate collaboration and reflection) and taking an interest in students. The ‘Perceived mentoring rewards’ index addresses the mentors’ perception of professional and personal development and gratification. See Table 4 for details on which items represents ‘Engaging mentoring approach’ and ‘Perceived mentoring rewards’. The ‘Perceived student resistance’ index represents mentors’ degree of insecurity related to whether students find the meetings meaningful, and mentors’ being disturbed if students appeared to be bored during the group meetings. Appendix 2 shows further details regarding the items included in each index.

All three indices were significantly associated with mentors’ satisfaction in the multilevel univariate linear analyses. See Table 2 for an overview of all predictor variables and p-values. Mentors who scored higher with regards to ‘Engaging mentoring approach’ and ‘Perceived rewards of mentoring’, were likely to score higher on satisfaction (β = 0.228, 95% CI 0.148, 0.310, and β = 0.428, 95% CI 0.330, 0.473, respectively). Furthermore, mentors who experienced lower levels of student resistance were likely to score higher with regards to satisfaction (β = -0.223, 95% CI -0.419, -0.240).

In addition to the three indices, the univariate analysis revealed that eight additional predictors were significantly associated with mentors’ satisfaction. These eight predictors were: how the mentors were recruited (voluntarily), years of experience as a mentor, items regarding mentor support (whether the expectations of the program were unclear or difficult to fulfil, and the mentors’ opinion about the quality of the mentor training and written material provided), and questions regarding students’ appreciation of the group meetings (if the mentors experienced that the students were enthusiastic and found the meetings valuable).

The results from the multilevel multivariate regression analysis are presented in Table 5. In this final model, after adjusting for all the variables mentioned, only two predictors remained significantly associated with mentors’ satisfaction: (1) the perception that the students found the group meetings valuable (β = 0.186, 95% CI 0.021, 0.351, *p* = 0.027) and (2) that the mentors experienced rewards of mentoring (β = 0.330, 95% CI 0.224, 0.437, *p* < 0.001). The ‘Perceived rewards’ index was the strongest predictor for mentors’ satisfaction both in the uni- and multivariate analyses.

**Table 5:** Associations between mentors’ satisfaction and predictor variables by multivariate multilevel linear regression analysis Dependent variable: Mentors’ satisfaction, derived from the question “If you consider the totality of your experience of being a mentor, how do you like it?”

Mentor characteristic or questionnaire item	Responses	β	*P*-value	95% CI
Intercept		3.950	< 0.001	[2.988, 4.911]
Age		9.813E-6	1.000	[0.080, 0.080]
Gender	Female = 1, Male = 2	0.134	0.115	[-0.033, 0.301]
Whether the mentors were recruited voluntarily or mandatory	1: I volunteered, 2: I was strongly urged, 3: It is mandatory in my job	-0.048	0.517	[-0.194, 0.098]
Years of experience as a mentor	0: <5 years, 1: 5–9 years, 2: =>10 years	0.003	0.948	[-0.103, 0.110]
The program’s expectations are unclear	1: Strongly disagree to 5: Strongly agree	-0.046	0.381	[-0.150, 0.058]
It is difficult to fulfil the program’s expectations	1: Strongly disagree to 5: Strongly agree	-0.042	0.408	[-0.143, 0.058]
Opinion about the quality of training provided to mentors	1: Very poor to 5: Very good	-0.003	0.971	[-0.145, 0.140]
Opinion about the quality of the written material provided to mentors	1: Very poor to 5: Very good	-0.085	0.225	[-0.224, 0.053]
The students seem to find the group meetings valuable*	1: Strongly disagree to 5: Strongly agree	0.186	0.027	[0.021, 0.351]
The students have lots of ideas for group discussions	1: Strongly disagree to 5: Strongly agree	0.037	0.436	[-0.056, 0.130]
‘Engaging mentor approach’	Factor containing six items that illustrates an engaging way to be a mentor	0.036	0.450	[-0.058, 0.129]
‘Perceived rewards’*	Factor containing six items that highlight potential rewards of being a mentor	0.330	< 0.001	[0.224, 0.437]
‘Perceived student resistance’	Factor containing three items that describe students’ resistance in the groups	-0.063	0.240	[-0.169, 0.043]

*p < 0.05

## Discussion

In the present study, group mentors at two Norwegian and one Canadian medical school seemed to be highly satisfied with their mentoring function in general. High satisfaction levels were significantly associated with mentors’ perceived rewards and their belief that students seemed to find the group meetings worthwhile.

Previous studies have shown that mentors value personal rewards such as an enhanced insight in the students’ thoughts and professional progress, as well as their own development [14, 32, 33]. This is aligned with the findings of this study; mentors who experience personal development, as well as gratifying relationships with the students, seem to be more satisfied in their role. Such “non-materialistic” rewards may be of higher importance for mentors’ satisfaction than pecuniary rewards, as we did not find any association between satisfaction and receiving additional financial compensation. Some well-known theories of satisfaction and motivation emphasize the impact of non-financial rewards, such as Self-Determination Theory (SDT). It suggests that intrinsic motivators such as autonomy, competence, and relatedness are crucial for enhancing intrinsic motivation, leading to higher satisfaction [34]. Financial incentives may be used to decrease “wear and tear”, though it seems not to be essential for mentors’ satisfaction [1, 13].

Previous studies on mentorships in medical education have suggested that learning activities should be highly structured and aligned with the overall curriculum [1, 3, 35]. In the current study, it is noteworthy that the design varied across the three schools. At UiT, every group meeting has predetermined topics with which mentors and mentees are expected to become acquainted and to discuss. This was in contrast with McGill University and UiB, where the meeting agenda was driven by students’ needs and interests and where preassigned content was relegated to a ‘back-up’ status. Interestingly, we found no significant association between group structuring and mentors’ satisfaction.

Mentors have an important facilitator role in group settings in that they encourage the mentees to share their personal and professional experiences in a safe environment [1, 19]. Some studies suggest that mentors with a more directive approach, one where the mentor responds to questions rather than fostering discussions in the group, may lead mentees to take on a passive role as spectators [5, 36]. This may impede reflective dialogues and professional development of both the mentors and mentees [1, 36]. As our findings suggest, it is likely that mentors feel more gratified if they experience that students are enthusiastic and find the group useful.

We created an index, representing mentors who perceive themselves as sharing, serving as role models and being group-oriented, and was inspired by previous research that attempted to describe desirable mentor approaches [6, 16, 18]. Somewhat surprisingly, in the multivariate analyses, we found no significant association between the ‘Engaging mentoring approach’ and mentors’ satisfaction. In contrast to previous studies, our findings suggest that whether mentors are satisfied in their role or not, is less dependent on their specific approach and more related to the group dynamics itself, as well as the mentors’ perceived rewards in their role.

Our findings have implications for our understanding of what might motivate mentors and thus how to sustain mentorship programs. We suggest that faculty development should focus on mentors’ experienced psycho-social rewards, as well as on strategies to establish competence in establishing well-functioning group dynamics. A recent review proposed that it is essential for mentors to undergo training that covers their role’s obligations [3]. Based on our study, there is reason to believe that by guiding mentors in group facilitation, relationships may be improved, ultimately leading to greater mentor satisfaction.

The findings of this study may not be surprising because ‘satisfaction’ and ‘rewards’ (regardless of their nature) are likely to be intertwined. However, it has clarified the situation. Financial rewards were not salient. To our knowledge, this is the first study to explore several predictors of group mentors’ satisfaction, while simultaneously controlling for socio-demographic variables and university-level effects.

### Study limitations

Although there are inherent limitations, the study has several strengths. We conducted it at three universities in two countries, where mentorship programs have been established for several years. Despite some differences (e.g. the methods for mentor recruitment, the organization of group meetings), this multi-centered study has provided a comprehensive overview of mentors’ experiences. The survey had acceptable response rates, and the panel of questions has given us the opportunity to show associations between the mentors’ level of satisfaction and several influential factors.

However, the findings should be interpreted considering certain limitations. As this was a cross-sectional study, we cannot use these data to infer causality [37]. The mentors’ responses were potentially influenced by additional factors that the survey instrument was not designed to explore in depth, such as the nature and content of faculty development, administration, and logistics, and the material or symbolic rewards or inconveniences of being a mentor. Another limitation is the fact that survey respondents may have been more positive about their mentoring role than non-respondents, from whom we have no information. Although some of the data were not continuous and showed a skewed distribution, we chose to analyze data with parametric methods. Several studies have shown that results of parametric analyses are robust and minimally sensitive to violations of assumptions of normality and type of scale [38, 39].

Future research should attempt to further explore the mentors’ perceptions of what constitutes well-functioning mentoring groups, as this seems to be important for mentors’ satisfaction. It should include prospective longitudinal studies, ideally multi-institutional, that examine the influence of group mentoring features on both mentors’ and mentees satisfaction on a long-term basis. Also, it would be valuable to study the extent of alignment between mentors’ and students’ perception on this matter.

## Conclusion

Functioning as a mentor for groups of medical students is seen as a satisfying and rewarding educational role. The findings of this study indicate that for group mentors’ overall satisfaction, it is highly important that they experience rewards including personal and professional development and gratifying relationships with the students. We propose that the mentors’ experienced psycho-social rewards, as well as competence in establishing well-functioning group dynamics, should be areas for faculty development. Guiding mentors in their roles as group facilitators may lead to improved relationships within their groups, which could potentially lead to greater mentor satisfaction and improved pedagogical quality of the group mentorship program.

### Supplementary Information

**Supplementary Material 1.**

**Supplementary Material 2.**

## Data Availability

Details regarding the survey sections and questions can be found in Appendix 1.
