# Supplementary material for: What makes mentors thrive? An exploratory study of their satisfaction in undergraduate medical education
Source: BMC Med Educ. 2024 Apr 4;24:372. doi: 10.1186/s12909-024-05344-y (PMC10996132; doi:10.1186/s12909-024-05344-y)
Supplement: Supplementary file 1 — Supplementary Material 1. [file 12909_2024_5344_MOESM1_ESM.docx]

## **Appendix 1 Survey categories, items, and responses**

| **Categories** | **Items and responses** |
| --- | --- |
| **Personal characteristics** | - Gender *(Male, female, I prefer not to answer)* - Age *(Below 40, 40-49, 50-59, 60+)* |
| **Professional characteristics** | - Where are you a mentor *(Tromsø, Bergen, McGill)* - If you have had a group in 2016-17, how many years has the group functioned *(not had – 4 years)* - Volunteered or mandatory *(volunteered – strongly urged – mandatory)* - Nature of current work *(mostly – some - not clinical)* - For how many years, in total, have you been a mentor for medical students *(free-text)* |
| **Satisfaction** | - If you consider the totality of your experience of being a mentor, how do you like it (*1 – I dislike to 5 – I like*) |
| **Mentor support** | Indicate your agreement with the following statements *(1 – strongly disagree to 5 – strongly agree + 6 – don’t know)*   - I find it unclear what the mentor program’s expectations are (i.e. the intended curriculum) - I find it difficult to fulfill the program’s expectations (i.e. the intended curriculum) - Currently, I find it difficult to invest in my mentoring function - What is your opinion about the quality of the training provided, including workshops and information meetings, to help mentors? *(1 – very poor to 5 – very good)* - What is your opinion about the quality of the written material on mentoring and the mentor program, provided to you as a mentor? *(1 – very poor to 5 – excellent)* - I rely strongly on the material and teaching methods provided by the university to structure the mentor meetings - I let the group decide how to use the time, without any preconceived plan - Other suggestions for training and information offered to mentors *(free-text)* |
| **Student perspective** | - Indicate your agreement with the following statements *(1 – strongly disagree to 5 – strongly agree + 6 – don’t know)*   - The students seem to find the group meetings worthwile   - The students in my group have lots of ideas for the group process and discussions |
| **Mentoring approach*** | - Indicate your agreement with the following statements *(1 – strongly disagree to 5 – strongly agree)*   - As a mentor I answer questions and provide knowledge   - As a mentor I share what it means to be a doctor   - As a mentor I listen to students without offering advice   - As a mentor I stimulate collaboration and relationships within the group   - As a mentor I am a role model for the students   - As a mentor I provide career counseling   - As a mentor I take an interest in students’ personal development   - As a mentor I share my experiences of doubt and uncertainty   - As a mentor I share my attitudes and judgments concerning values and dilemmas in medicine |
| **Rewards*** | - Indicate the importance of the following rewards *(1 – not important to 5 – very important)*   - I learn a lot from discussing with students   - The preparation and orientation offered to all mentors gives me new knowledge   - The relationships with students are gratifying   - Mentoring makes me more proud of being a physician   - Mentoring allows me to explore what it means to be a “good doctor”   - Mentoring provides financial rewards - Indicate your agreement with the following statements *(1 – strongly disagree to 5 – strongly agree + 6 – don’t know)*   - Being a mentor has helped me become better at what I do professionally - Other aspects of mentoring that you find rewarding *(free-text)* |
| **Student resistance*** | - Indicate your agreement with the following statements *(1 – strongly disagree to 5 – strongly agree + 6 – don’t know)*   - Students participate because it is mandatory, not because they appreciate its value   - It is difficult to know whether students in my/our group find the meetings worthwhile - It is disturbing when students seem to dislike or be bored during meetings |
| **Mentoring experiences** | - Willing to be a mentor again for a new group *(1 – definitely not to 5 – definitely yes)* - Other comments regarding the mentoring experience *(free-text)* |
| **Interesting topics** | - Indicate how interesting the following topics are for discussion in your group (*1 – completely uninteresting to 5 – very interesting)*   - Clinical communication   - Career planning   - Students’ clinical experiences   - Students’ experiences in medical school   - Students’ private experiences   - How the mentor group works for the participants   - Physicians’ wellness issues   - Medical students’ wellness issues   - Clinical skills training   - Ethical dilemmas   - Health politics   - Poverty and health   - Clinical reasoning   - Issues of empathy   - Patient-centered medicine   - Suffering and sickness |
| **Time and/or attention paid to topics** | - How much time and/or attention has been paid to each topic so far *(1 – not discussed to 3 – discussed a lot)*   - Clinical communication   - Career planning   - Students’ clinical experiences   - Students’ experiences in medical school   - Students’ private experiences   - How the mentor group works for the participants   - Physicians’ wellness issues   - Medical students’ wellness issues   - Clinical skills training   - Ethical dilemmas   - Health politics   - Poverty and health   - Clinical reasoning   - Issues of empathy   - Patient-centered medicine   - Suffering and sickness - Indicate other topics that have been important *(free-text)* |
| **Co-mentoring** | - Do you have a co-facilitator or co-mentor? *(Senior student, physician, others or no)* - If you have a co-mentor, how different is he or she from you on the following characteristics? *(free-text)* - My co-mentors’ gender *(1 – same, 2 – different)* - My co-mentors’ age *(same, more than five years older or younger)* - My co-mentors’ way of being a mentor *(1 – roughly the same, 2 – clearly different: free-text)* - My co-mentor's experience as a physician *(1 – lower level, 2 – roughly same level, 3 – higher level than me)* - My co-mentor's experience as teacher *(1 – lower level, 2 – roughly same level, 3 – higher level than me)* - Shared responsibility of mentoring in a fair way *(1 – yes, 2 – too much has fallen on me, 3 – too much has fallen on my partner)* - Indicate your agreement with the following statements (*(1 – strongly disagree to 5 – strongly agree)*   - I would generally prefer to work in a pair rather than as a single mentor   - Being in a pair gives me more flexibility in scheduling meetings   - Being in a pair provides more material and issues for group discussions   - I like very much to work with my co-mentor   - My co-mentor and I have very different roles in the group   - In general, the program would benefit if mentor pairs where changed halfway through the mentorship program   - I would not have volunteered as a mentor if it meant leading the group alone   - Being in a pair enhances relationship building with students   - Being in a pair enhances the richness of the mentoring experience   Students as co-mentors (Canadian mentors)   - Has your relationship with your co-mentor developed into a resource for you, in any of the following ways? *(1 – yes, 2 – no, 3 – not applicable)*   - In clinical work   - Creating network, providing contacts   - Personal development   - In research   - In teaching   - In mentoring - For my functioning as a mentor, my student co-mentor has represented *(1 – a very negative contribution to 5 – a very positive contribution)* - Indicate your agreement with the following statements *(1 – strongly disagree to 5 – strongly agree)*   - Helped me out with logistics and organization of the mentor meetings   - Helped me understand and connect with the students   - Challenged my authority in the group   - Helped me with ideas and suggestions for reflection   - Had difficulties understanding his or her role as a mentor for the younger students - Other comments on the experience of having a student co-mentor *(free-text)*   Physicians as co-mentors (Norwegian mentors)   - Working as a duo would have the following advantages *(1 – strongly disagree to 5 – strongly agree)*   - There would be more flexibility in scheduling meetings   - It would provide more material and issues for group discussions   - It would enhance relationship building and connecting with students   - It would enhance the richness of the experience and contribute to my learning - If you were to choose the best co-mentor arrangement for you, which of the following would you prefer *(1 – not prefer to have, 2 – senior student, 3 – physician, 4 – no preference, 5 – no clear opinion)* |

*=Items that were included in factor analyses
