# Supplementary material for: What makes mentors thrive? An exploratory study of their satisfaction in undergraduate medical education
Source: BMC Med Educ. 2024 Apr 4;24:372. doi: 10.1186/s12909-024-05344-y (PMC10996132; doi:10.1186/s12909-024-05344-y)
Supplement: Supplementary file 2 — Supplementary Material 2. [file 12909_2024_5344_MOESM2_ESM.docx]

**Appendix 2 Factor analyses results for mentoring approach, perceived rewards of mentoring and perceived student resistance**

## 1 Exploratory factor analysis results for items concerning mentoring approach

| **Mentoring approach** | Factor Loadings | | |
| --- | --- | --- | --- |
|  | 1* | 2 | 3 |
| As a mentor I answer questions and provide knowledge** | -0.005 | 0.803 | -0.114 |
| As a mentor I share what it means to be a doctor | 0.673 | 0.284 | -0.229 |
| As a mentor I listen to students without offering advice** | -0.059 | -0.117 | 0.863 |
| As a mentor I stimulate collaboration and relationships within the group | 0.666 | -0.230 | 0.163 |
| As a mentor I am a role model for the students | 0.797 | 0.080 | 0.043 |
| As a mentor I provide career counselling** | 0.217 | 0.684 | 0.088 |
| As a mentor I take an interest in students’ personal development | 0.361 | 0.396 | 0.535 |
| As a mentor I share my experiences of doubt and uncertainty | 0.549 | 0.333 | 0.240 |
| As a mentor I share my attitudes and judgments concerning values and dilemmas in medicine | 0.648 | 0.251 | 0.018 |
| Eigenvalues and % of variance | 2.92 (32.38%) | 1.245 (13.83%) | 1.061 (11.80%) |
| *= Items that comprise the ‘Engaging mentoring approach’, except the items that were removed  **= Items that were removed due to minimal loading to the factor | | | |

## 2 Exploratory factor analysis results for items concerning perceived rewards of mentoring

| **Perceived rewards** | Factor Loadings | |
| --- | --- | --- |
|  | 1* | 2 |
| Being a mentor has helped me become better at what I do professionally | 0.726 | 0.166 |
| I learn a lot from discussing with students | 0.729 | 0.210 |
| The preparation and orientation offered to all mentors gives me new knowledge | 0.558 | 0.506 |
| The relationships with students are gratifying | 0.835 | -0.163 |
| Mentoring makes me more proud of being a physician | 0.832 | 0.059 |
| Mentoring allows me to explore what it means to be a “good doctor” | 0.813 | 0.138 |
| Mentoring provides financial rewards** | -0.014 | 0.919 |
| Eigenvalues (% of variance) | 3.560 (50.86%) | 1.082 (15.46%) |
| *= Items that comprise the ‘Perceived rewards’, except the item that were removed  **= Item that were removed due to minimal loading to the factor | | |

## 3 Factor analysis results for items concerning perceived student resistance

| **Perceived student resistance** | Factor Loadings |
| --- | --- |
|  | 1* |
| Students participate because it is mandatory, not because they appreciate its value | 0.854 |
| It is difficult to know whether students in my/our group find the meetings worthwhile | 0.827 |
| It is disturbing when students seem to dislike or be bored during meetings | 0.655 |
| Eigenvalues (% of variance) | 1.842 (61.41%) |
| *= Items that comprise the ‘Perceived student resistance’ | |
